# Changing Food in a Changing World: Assessing Compliance to Insects, Cultivated Meat, and Soil-Less Products Among Italian Undergraduates

**DOI:** 10.3390/nu17050909

**Published:** 2025-03-05

**Authors:** Francesca Gallè, Federica Valeriani, Jole Del Prete, Patrizia Calella, Annalisa Bargellini, Aida Bianco, Lavinia Bianco, Salvatore Borzì, Anastasia Cataldo, Maria Eugenia Colucci, Laura Dallolio, Osvalda De Giglio, Chiara de Waure, Gabriella Di Giuseppe, Pasqualina Laganà, Giuseppe La Spada, Francesca Licata, Isabella Marchesi, Alice Masini, Maria Teresa Montagna, Christian Napoli, Stefania Oliva, Giovanna Paduano, Stefania Paduano, Cesira Pasquarella, Concetta Paola Pelullo, Michela Persiani, Ivano Pindinello, Vincenzo Romano Spica, Rossella Sacchetti, Giacomo Scaioli, Concetta Arianna Scicchitano, Roberta Siliquini, Francesco Triggiano, Licia Veronesi, Carmela Protano

**Affiliations:** 1 Department of Medical, Movement and Wellbeing Sciences, University of Naples “Parthenope”, 80133 Naples, Italy; patrizia.calella@uniparthenope.it (P.C.); concettapaola.pelullo@uniparthenope.it (C.P.P.); 2 Department of Movement, Human, and Health Sciences, University of Rome “Foro Italico”, 00135 Rome, Italy; federica.valeriani@uniroma4.it (F.V.); vincenzo.romanospica@uniroma4.it (V.R.S.); 3 Department of Public Health and Infectious Diseases, Sapienza University of Rome, 00185 Rome, Italy; jole.delprete@uniroma1.it (J.D.P.); salvatore.borzi@uniroma1.it (S.B.); ivano.pindinello@uniroma1.it (I.P.); carmela.protano@uniroma1.it (C.P.); 4 Department of Biomedical, Metabolic and Neural Sciences, University of Modena and Reggio Emilia, 41125 Modena, Italy; annalisa.bargellini@unimore.it (A.B.); isabella.marchesi@unimore.it (I.M.); stefania.paduano@unimore.it (S.P.); 5 Department of Medical and Surgical Sciences, University of Catanzaro “Magna Græcia”, 88100 Catanzaro, Italy; a.bianco@unicz.it (A.B.); a.scicchitano@unicz.it (C.A.S.); 6 Department of Medical Surgical Sciences and Translational Medicine, Sapienza University of Rome, 00189 Rome, Italy; lavinia.bianco@uniroma1.it (L.B.); christian.napoli@uniroma1.it (C.N.); stefania.oliva@uniroma1.it (S.O.); 7 National Institute for Health, Migration and Poverty (NIHMP), 00153 Rome, Italy; 8 Department of Public Health Sciences and Pediatrics, University of Turin, 10126 Turin, Italy; anastasia.cataldo@unito.it (A.C.); giacomo.scaioli@unito.it (G.S.); roberta.siliquini@unito.it (R.S.); 9 Department of Medicine and Surgery, University of Parma, 43125 Parma, Italy; mariaeugenia.colucci@unipr.it (M.E.C.); ira.pasquarella@unipr.it (C.P.); licia.veronesi@unipr.it (L.V.); 10 Department of Biomedical and Neuromotor Science, Alma Mater Studiorum, University of Bologna, 40126 Bologna, Italy; laura.dallolio@unibo.it (L.D.); michela.persiani3@unibo.it (M.P.); 11 Interdisciplinary Department of Medicine, University of Bari Aldo Moro, 70124 Bari, Italy; osvalda.degiglio@uniba.it (O.D.G.); mariateresa.montagna@uniba.it (M.T.M.); francesco.triggiano@uniba.it (F.T.); 12 Department of Medicine and Surgery, University of Perugia, 06132 Perugia, Italy; chiara.dewaure@unipg.it; 13 Department of Experimental Medicine, University of Campania “Luigi Vanvitelli” Naples, 80138 Naples, Italy; gabriella.digiuseppe@unicampania.it (G.D.G.); giovanna.paduano@unicampania.it (G.P.); 14 Department of Biomedical and Dental Sciences and Morphofunctional Imaging, University of Messina, 98122 Messina, Italy; plagana@unime.it (P.L.); giuseppe.laspada@unime.it (G.L.S.); 15 Department of Health Sciences, University of Catanzaro “Magna Græcia”, 88100 Catanzaro, Italy; f.licata@unicz.it; 16 Department of Translational Medicine, Amedeo Avogadro University of Eastern Piedmont, 13100 Novara, Italy; alice.masini@uniupo.it; 17 Department of Education Studies “Giovanni Maria Bertin”, Alma Mater Studiorum, University of Bologna, 40126 Bologna, Italy; rossella.sacchetti@unibo.it

**Keywords:** novel food, sustainability, undergraduates, propensity

## Abstract

**Background/Objectives**: In recent decades, the need for sustainable alternatives to traditional foods for the global population has become urgent. To this aim, edible insects, cultivated meat, and vegetables produced through soil-less farming have been proposed. This cross-sectional study was aimed at exploring willingness to eat these novel foods and its possible correlates in young Italian adults. **Methods**: An electronic questionnaire was administered to the student populations of 13 universities throughout the Italian territory. **Results**: The results show that insects and cultivated meat were widely acknowledged as possible food, while soil-free cultivation seems to be less known. Indeed, the percentage of participants who have heard of insects, cultivated meat, and soil-free cultivation was respectively equal to 91.5%, 84.7%, and 32.9%. However, the majority of respondents were uncertain about the sustainability of all the proposed products (52.6% for insects, 39.5% for cultivated meat, and 58.0% for soil-free cultivation, respectively), and the propensity to try and eat insects (9.5%) was lower than that declared for synthetic meat (22.8%) and products from soil-free cultivation (19.2%). However, the regression analysis showed that the propensity to eat these foods regularly is positively related to the confidence in their sustainability (*p* < 0.001). Willingness to try each of the proposed foods positively correlated with that declared for the others (*p* < 0.001). **Conclusions**: These findings highlight the need to implement interventions aimed at increasing awareness about the use of these products as alternatives to less sustainable foods and the importance of identifying consumer groups to which these interventions should be addressed.

## 1. Introduction

Due to the increase in the world’s population, it is expected that the global demand for meat will grow by more than two-thirds in 2050 [1]. However, evidence shows that intensive livestock farming is responsible for a great amount of greenhouse gas emissions [2] and is associated with biodiversity loss and with the degradation and depletion of land, soil, and water resources [3]. At the same time, one-third of the global land area is being used for agricultural production to feed the world population [4].

Therefore, there is a need for food that can represent a valuable source of nutrients for a growing population while addressing environmental issues.

Among the proposed solutions are some “novel foods”, namely edible insects, cultivated meat, and vegetables produced through soil-less farming, which were not consumed in Europe in the past and are gaining increasing attention. While some of these novel foods, such as hydroponically grown vegetables, are already available on the market in certain regions, others, like cultivated meat, are still in the development phase and not yet widely commercialized [5]. The adoption of these foods on a larger scale depends on technological advancements, regulatory approvals, and consumer acceptance. Consequently, discussions often focus on their potential future consumption rather than their current widespread use [6]. Insect-based food refers to insect species used for human consumption, as a whole or as an ingredient in processed food products such as pasta or snacks. Insects are considered a promising source of animal protein alternative to traditional food, with a reduced environmental impact [7]. The terms “cultivated meat” or “synthetic meat” are used to describe the muscle fibers produced by introducing muscle cells from donor bovine animals to a culture medium, where they proliferate under controlled conditions [8]. Soil-less farming systems include several technologies that have been gradually expanding because of their high crop yield and reduced land use [9]. Specifically, in hydroponics, farming plants are grown with roots submerged in aqueous nutrient solutions with inert substrates, while in aquaponics farming, plants are supplied with nutrient-dense aquaculture water, and in aeroponic farming, roots are suspended in the air and misted with nutrient-dense water [10].

The literature shows that several food, societal, and individual characteristics, as well as the correct knowledge of sustainable products, are related to the propensity to use sustainable foods. In a cross-sectional study performed among people from 14 countries, a moderate knowledge regarding the sustainability of edible insects was registered, and it was found to be related to different sociodemographic and behavioral characteristics [11].

In the study by Giezenaar and colleagues, about half of the respondents from New Zealand, mainly men and older adults, were aware of cultivated meat. Their willingness to consume this food was associated with their awareness [12]. Similarly, Silva and co-authors showed that consumers’ intention to buy cultivated meat is related to its sustainability appeal and with the awareness of the production process [13]. Furthermore, in a study performed by Leung and colleagues in Singapore, a positive relationship between psychological well-being and willingness to consume cultivated meat was shown [14]. As for soil-less cultivations, in the study performed on a sample of Swedish adults by Spendrup and collaborators, less than half of the respondents were aware of hydroponics, with education as a predictor [15]. Instead, the attitude to use them was positively related to age and to not having heard of hydroponic cultivations before [16]. The review by Zhou and co-authors reported that attitude towards soil-free products is mostly related to income, education, and knowledge of consumers, so as with sensory properties, sustainability, and growing conditions of products [10]. Furthermore, consumer awareness, environmental concern, and health consciousness were found to positively influence consumers’ willingness to buy hydroponic vegetable products [17].

In the perspective of their spread as sustainable alternatives to traditional foods, it is important to analyze the acceptance of consumers towards novel foods in order to identify those categories of individuals who are less ready to use them and provide them with correct information.

In the last few years, several studies have been conducted throughout different populations and cultures to assess the propensity to consume insects and synthetic meat. However, to the authors’ knowledge, no studies have been performed so far in Italy to explore people’s awareness about soil-free products. Furthermore, it could be useful to specifically analyze the attitude of young adults towards novel food because they represent possible future consumers. Therefore, this study was aimed at exploring willingness to eat vegetables from soil-free cultivation, insects, and cultivated meat in young adults from our whole country, highlighting possible correlations among these attitudes. In order to identify possible predictors of hesitancy towards these foods, possible associations with sociodemographic characteristics and habits of participants were also analyzed.

## 2. Materials and Methods

### 2.1. Study Design and Participants

This cross-sectional study took place between February and October 2024 and involved undergraduate students from 13 universities, distributed across different regions of Italy.

The study employed a digital questionnaire designed and hosted on the EU Survey platform, a tool funded by the European Commission and approved by the European Union for its robust data protection features (available at https://ec.europa.eu/eusurvey/, accessed on 8 February 2024). The questionnaire was distributed via a secure link, which was shared with students during their academic courses after an initial presentation explaining the aims and procedures of the study and asking them to further spread the link to their fellow students.

The inclusion criteria were being adult and undergraduate students at one of the participating universities. Questionnaires that were incomplete or demonstrated clear evidence of unreliable responses, such as random patterns or uniformly repeated answers, were excluded from the final analysis to ensure data integrity and validity.

The protocol of the study received ethical approval from the Ethics Committee of the University “Foro Italico” of Rome (approval number 179/2024).

The study adhered strictly to the principles outlined in the Declaration of Helsinki, ensuring ethical rigor at every stage. Informed consent was obtained digitally, with participants explicitly agreeing to the use of their anonymized data solely for academic purposes. Anonymity was fully guaranteed in compliance with Italian data protection regulations, with all data processed in an aggregated and anonymized form.

### 2.2. Questionnaire

In order to assess propensity towards the proposed foods and to identify possible correlates, data were collected through a structured questionnaire, which was divided into two main sections: the first aimed to explore the demographic characteristics and lifestyle of participants, and the second focused on their attitudes and propensity towards the consumption of the three categories of novel food.

Specifically, the first section included questions on gender, age, nationality, parental education level, the university they were enrolled in, and the type of degree program they were attending. Further, this section explored the presence of chronic health conditions, dietary patterns (“No diet regimen”, “Mediterranean diet”, and “Other type of diet”), and the use of dietary supplements (“Yes” or “No”).

The questions included in the second part of the questionnaire were developed to comprehensively assess participants’ familiarity with, attitudes toward, and propensity to consume the three categories of novel foods. To guarantee participants understood the context, each category was introduced with a brief description. Edible insects were defined as those species of insects that are commercially available for consumption, either whole or processed into products like flour. The term synthetic meat referred to meat cultivated in vitro from animal cells derived from chickens, cows, or pigs. Soil-free cultivation products were described as plants grown in controlled environments without the use of traditional soil, relying on substrates like water, minerals, or alternative materials such as coconut fiber or clay. This introduction allowed participants to answer subsequent questions even if they did not know the proposed foods. To ensure the robustness of the design and the relevance of the questions posed in this section, we drew inspiration from established methodologies used in prior studies on consumer behavior toward novel and unconventional foods [15]. We adapted the structure and response formats to suit the context of our research, ensuring consistency across the three food categories while maintaining flexibility in order to capture nuanced insights specific to our study population. The design was further tailored to address sustainability, willingness to try and consume, and price sensitivity, factors identified in the literature as critical for understanding consumer acceptance of novel foods.

Then, five questions were posed for each novel food category to assess whether participants had ever heard of the specific food before this study (with possible responses being “Yes” or “No”), whether they considered its consumption to be more sustainable compared to conventional alternatives (“Much less”, “Less”, “Not sure”, “More” or “Much more”), their willingness to try it (assessed on a 5-point scale ranging from “Absolutely no” to “Absolutely yes”, whether they were willing to eat it regularly (“Yes” or “No”), and how much they would be willing to pay for such products (5 replies from “Much less” to “Much more”). The full questionnaire is available as a Supplementary File S1.

### 2.3. Statistical Analysis

Continuous quantitative variables were described by the mean, standard deviation, median, and interquartile range of values, while categorical variables were summarized through absolute and relative frequencies expressed as percentages for each category. The “geographical area” variable was derived from participants’ university responses and then classified into three categories: North, Center, and South Italy. Similarly, the “study area” variable was created by categorizing responses regarding the participants’ course of study into two groups: medical/health-related fields and other fields.

The variable “Diet regimen” was categorized into two groups: “Mediterranean diet” and “Other type of diet”. The latter category encompassed no diet regimen or calorie-restricted diet, diet adapted to specific health conditions, vegetarian or vegan diet, and other dietary types.

A univariate analysis was performed to identify possible statistically significant differences regarding the collected variables between individuals who were likely to consume novel foods regularly and those who were not. For categorical variables, the chi-square test was applied. The Mann–Whitney U test was employed for numerical variables with non-normal distribution, while the T-test was used for the numerical variable with a normal distribution.

In order to identify possible variables influencing the propensity for regular consumption, a multivariate analysis was conducted by creating 3 multiple regression models, one for each novel food category investigated. Willingness to consume these foods regularly was considered as the outcome, while sociodemographic and health characteristics, dietary habits, knowledge of each novel food, and beliefs about its sustainability with respect to traditional alternatives (categorized as less = “much less”/“less”/“uncertain” and more = “more”/“much more”) were used as independent variables. Age and gender were included in all models regardless of the significance of their association. The analysis evaluated multicollinearity, the presence of unnecessary variables using the Likelihood Ratio Test, and the calibration and discrimination of the models to develop the final models. Associations were assessed by calculating odds ratios (ORs) and their corresponding 95% confidence intervals (95%CI).

Furthermore, a Spearman’s correlation analysis was performed to assess possible associations among propensity to consume the three foods. To this aim, a 0–4 score was attributed to the answers to the question related to the willingness to try each food.

Significance was set at *p* = 0.05. The database management and statistical analyses were performed using STATA, version 18 (StataCorp LLC, College Station, TX, USA).

## 3. Results

A total of 1790 questionnaires were obtained from the study participants. Only two of them (0.1) were excluded due to unreliable answers. Table 1 shows the main characteristics of the 1788 participants in the final sample.

Briefly, the sample had a mean age of about 25 years and showed high proportions of females and healthy subjects. Almost the entire sample consisted of Italians, and Southern Italy was the most represented geographic area, while the medical/health sector was the most common area of study. The majority of the sample did not follow a specific dietary model and did not use dietary supplements.

With regard to the knowledge of the novel foods under study, participants had heard mainly of edible insects and synthetic meat compared to soil-free products (Table 2).

The majority of the samples were uncertain about the higher sustainability of the three novel foods with respect to the corresponding traditional foods. As for their propensity to try these products, higher willingness was registered towards synthetic meat and soil-free cultivation products than towards insects. The same finding was found for the willingness to use them regularly. More than 70% and 60% of respondents declared their willingness to pay less for insects and synthetic meat, respectively, while about 44% of them would like to pay the same money to buy soil-free and conventional cultivation products.

In the univariate analyses (Supplementary File S2) the propensity to consume edible insects significantly differed among groups defined by age, gender, nationality, parents’ level of education, geographic area, diet, and opinion regarding food sustainability; the propensity to consume synthetic meat differed among groups defined by gender, parents’ level of education, geographical area, previous knowledge and opinion about sustainability; the propensity to consume soil-less products differed among groups defined by age, parents’ level of education, geographic area, diet, knowledge and opinion regarding sustainability. Therefore, these variables were included in the regression analyses.

Table 3 shows the results of the regression analyses for the three types of food conducted by including those variables who showed significant differences in the univariate analyses. As for sociodemographic and behavioral variables, in the regression model studying the propensity to consume edible insects as the dependent variable, male gender, non-Italian nationality, mother’s education, studying at a university in Central Italy, and having a Mediterranean diet were found to be positively associated with the outcome; male gender and father’s educational level were associated with the propensity to consume synthetic meat; the propensity to consume vegetables from soil-free farming was found to be positively associated with age, father’s education and studying at a university in Northern Italy. Finally, being aware of synthetic meat and soil-less products was found to be related with willingness to eat them and being confident in their sustainability was found to be associated with willingness to eat all the three foods.

Spearman’s correlation analysis showed that the propensity towards each type of proposed food was positively related to the propensity towards the other proposed foods (*p* < 0.01).

## 4. Discussion

This study was aimed at assessing the propensity towards specific sustainable foods among Italian young adults and at identifying factors possibly associated with it. Our findings show that insects and cultivated meat were widely acknowledged as possible food, while soil-free cultivation seems to be less known. However, the majority of respondents were uncertain about the sustainability of all the proposed foods, and the propensity to try and eat insects was lower than that declared for synthetic meat and products from soil-free cultivation. Furthermore, the greatest part of our sample would be willing to pay less for insects and cultivated meat than for traditional food and thought that products from soil-free cultivation should cost the same as common vegetables. These findings partially agree with those reported by previous investigations.

As for insects, the higher proportion of respondents who had heard of them as possible food in spite of their recognized environmental impact and the low propensity to eat them reflects the findings of a similar study performed by Lorini and co-workers in a sample of undergraduates from an Italian university [18] and those by Wilkinson and colleagues, who showed that 68% of their Australian consumers had previously heard of entomophagy, but only 21% had eaten insects [19]. However, our results showed lower willingness rates than other studies. In a survey involving 3556 students from three Italian universities, a mean score of 4.6 out of 7 was found regarding the willingness to eat food containing insects [20], while in a recent study by Vanutelli and collaborators, an average intention of 5.92 out of 10 to try insects was found among Italian undergraduates [21]. This could be due to the different type of questionnaire used in our study; it should also be considered that, being tested on three types of food, participants unconsciously made an unwanted comparison and gave preference to synthetic meat and vegetables from soil-less cultivation with respect to insects.

However, with 31.6% of our sample who were ready to try synthetic meat and only 7.9% willing to eat it regularly, even the propensity for this type of food was lower than that reported in other studies. In 2020, Bogueva and Marinova found that more than 70% of their sample of young Australian adults were not willing to accept this food, although it was identified as a sustainable alternative to farmed meat [22]. In the study by Franceković and collaborators, performed among 2007 respondents from Croatia, Greece, and Spain, 47% of the sample had not heard of cultivated meat, 47% were willing to try it, and 41% would pay the same money as for conventional meat [23]. As for Italy, the study by Liu and colleagues, which involved Italian, Portuguese, and Spanish consumers, reported a proportion of respondents who were ready to try synthetic meat equal to 66%, even though the Italian group showed lower willingness values than the others [17]. Furthermore, the study by Mancini and Antonioli reported that more than half of their sample of Italian consumers (54%) were willing to try cultivated meat [24].

With regard to the variables possibly associated with the willingness to eat these foods, some common elements emerged from the regression models. Male gender in particular seems to be positively related to the propensity to eat all the types of proposed foods, and belonging to a university from Central or Northern Italy was found to be related to willingness to consume insects and soil-less grown vegetables, respectively. Similarly, males were found to be more ready to eat insects and cultivated meat in previous studies [17,20,23]. The geographical associations highlighted by our study are in line with previous research [22] and may be explained with cultural and behavioral differences among the three geographical subgroups, which we observed in previous studies [25,26]. Probably, these differences were also responsible for the lower attitude towards insects and cultivated meat shown by our sample in comparison with the other studies performed in Italy, which involved a lower number of Italian universities [18,20,21].

Furthermore, a higher father’s educational level was found to be associated with willingness to eat synthetic meat and soil-less grown products in the regression models. Some studies have previously shown that consumers’ educational level is associated with acceptance of novel foods [24,27]. Since our sample involved only undergraduates, we chose to include the parents’ education as an indicator of participants’ socioeconomic status. Therefore, the association we found could indicate that individuals from lower socioeconomic status represent a population group to which information campaigns should be addressed. The inverse relationship found between the propensity for soil-less crops and mothers’ education highlights the need for further analyses on these variables.

Following a Mediterranean diet regimen was related to a propensity to consume insects. This is in contrast with the previous literature, which showed how the attachment to culinary tradition affects the dietary behaviors of the Italian population [28]. After all, the association between non-Italian nationality and propensity towards entomophagy seems in line with this aspect.

In line with previous studies, we also found that having heard of the proposed foods and acknowledging them as possible sustainable alternatives to traditional foods are associated with the propensity to consume the proposed foods [12,13,16]. Only the willingness to introduce insects in the diet was not found to be related to having heard of them, suggesting that other variables may play a greater role in determining this specific propensity. These data generally confirm the relationship between knowledge and attitude towards new sustainable food choices and underline the importance of spreading correct information in this context.

Finally, according to the findings by Spendrup and co-authors, age was positively related to the propensity towards vegetables from soil-less farming [15].

In addition, the correlation we found among the propensity levels, despite the differences among the proposed foods, suggests the presence of a general hesitancy towards novel food in our sample. This result deserves further investigations.

Some limitations should be acknowledged when interpreting these findings. First, although several universities were enrolled throughout the whole Italian territory, their involvement was based on a convenience method, and many participants attended courses in the medical-health care area, and these could affect the representativeness of the sample. Moreover, findings regarding the undergraduate population may not correspond to what concerns the general young adult population. Another possible cause of non-representativeness is related to the fact that persons presenting a negative approach to the new foods could be expected to be more reluctant to take part in the study, but this represents an intrinsic limitation of a study based on voluntary participation. Furthermore, the spread of the electronic questionnaire used in the study was not controlled and could not have allowed it to reach the whole student population of the universities involved. The reliability of the information collected hinges upon the thoroughness of participants’ answers. However, it should be considered that the digital format of the questionnaire allowed students to participate voluntarily at their convenience, providing a flexible and accessible means of data collection while maintaining the highest standards of data security and confidentiality.

## 5. Conclusions

In conclusion, although satisfying levels of propensity towards sustainable foods such as vegetables from soil-less cultivation were found in our sample, it seems that high hesitancy towards entomophagy and eating cultivated meat still persists. Above all, the inconsistency between the spread knowledge of investigated foods and the uncertainty about their sustainability, and the association found between propensity and beliefs in their sustainability, highlight the need to implement interventions aimed at increasing awareness about their employ as alternatives to less sustainable foods and the importance of identifying consumer groups to which these interventions should be addressed.

## Figures and Tables

**Table 1:** Characteristics of the participants (n = 1788).

Variable	Value
Age (years)	
mean value ± SD	24.79 ± 6.96
median value (interquartile range)	22 (7)
range	18–68
Gender n (%)	
females	1250 (69.91)
males	518 (28.97)
not specified	20 (1.12)
Nationality n (%)	
Italian	1728 (96.64)
other	60 (3.36)
Mother’s educational level n (%)	
primary school	479 (26.79)
secondary school	852 (47.65)
degree or postgraduate education	457 (25.56)
Father’s educational level n (%)	
primary school	595 (33.28)
secondary school	854 (47.76)
degree or postgraduate education	339 (18.96)
Geographical area n (%)	
North	660 (39.22)
Center	256 (15.21)
South	767 (45.57)
Study area n (%)	
medical-health care area	1218 (68.12)
other	570 (31.88)
Chronic disease n (%)	
yes	191 (10.68)
no	1538 (86.02)
Diet regimen n (%)	
no dietary regimen	755 (42.23)
Mediterranean diet	620 (34.68)
other diet	413 (23.10)
Use of nutritional supplements n (%)	
yes	714 (39.93)
no	1074 (60.07)

**Table 2:** Knowledge and propensity towards the examined novel foods declared by participants.

Variable	Edible Insects n (%)	Synthetic Meat n (%)	Soil-Free Cultivation Products n (%)
Ever heard of this food before this study			
yes	1636 (91.50)	1515 (84.73)	588 (32.89)
no	152 (8.50)	273 (15.27)	1200 (67.11)
Considering this food sustainable compared to conventional food			
much less	204 (11.41)	198 (11.07)	160 (8.95)
less	119 (6.66)	206 (11.52)	137 (7.66)
uncertain	941 (52.63)	706 (39.49)	1037 (58)
more	374 (20.92)	441 (24.66)	341 (19.07)
much more	150 (8.39)	237 (13.26)	113 (6.32)
Willing to try this food			
definitely no	595 (33.28)	287 (16.05)	127 (7.1)
probably no	364 (20.36)	193 (10.79)	104 (5.82)
unsure	263 (14.71)	268 (14.99)	531 (29.7)
probably yes	396 (22.15)	632 (35.35)	683 (38.2)
definitely yes	170 (9.51)	408 (22.82)	343 (19.18)
Willing to eat regularly this food			
Yes	141 (7.89)	344 (19.24)	472 (26.40)
No	1647 (92.11)	1444 (80.76)	1316 (73.60)
Willing to pay for this food compared to conventional food			
much less	982 (54.92)	551 (30.82)	337 (21.09)
less	519 (29.03)	590 (33)	532 (29.75)
same	260 (14.54)	514 (28.75)	780 (43.62)
more	17 (0.95)	126 (7.05)	93 (5.2)
much more	10 (0.56)	7 (0.39)	6 (0.34)

**Table 3:** Regression models for the willingness to eat regularly the three types of food examined. Only significant associations are shown.

	Willingness to Eat Regularly the Novel Food Odds Ratio (95% Confidence Interval)		
Independent Variable	Edible Insects	Synthetic Meat	Soil-Free Cultivation Products
Gender			
Female (reference)	1.00	1.00	1.00
Male	2.01 (1.42–2.84) **	1.48 (1.14–1.93) **	1.05 (0.81–1.38)
Age	1 (0.97–1.03)	1.00 (0.98–1.03)	1.02 (1.00–1.04) *
Nationality			
Italian (reference)	1.00		
Other	3.04 (1.37–6.76) *		
Mother’s educational level primary school (reference) secondary school degree or postgraduate education			1.00 0.67 (0.50–0.89) ** 0.94 (0.67–1.32)
Father’s educational level primary school (reference) secondary school degree or postgraduate education		1.00 1.41 (1.06–1.88) * 1.94 1.38–2.71 **	1.00 1.40 (1.07–1.84) * 1.56 (1.09–2.23) *
Geographical area			
South (reference)	1.00		1.00
Center	2.23 (1.30–3.83) **		1.06 (0.70–1.59)
North	1.47 (0.91–2.35)		1.61 (1.20–2.15) **
Diet regimen			
Other diet (reference)	1.00		
Mediterranean diet	1.50 (1.02–2.21) *		
Having heard of this food			
no (reference)		1.00	1.00
yes		3.75 (1.85–7.62) **	2.78 (2.13–3.62) **
Considering this food sustainable compared to conventional food			
less (reference)	1.00	1.00	1.00
not sure	1.13 (0.52–2.45) **	3.23 (1.56–6.69) *	1.85 (1.19–2.86) *
more	7.79 (3.82–15.90) **	29.08 (14.70–57.51) **	17.24 (11.02–26.98) **

* *p* < 0.05; ** *p* < 0.01.

## Data Availability

The raw data supporting the conclusions of this article will be made available by the authors on request.

## Supplementary Materials

The following supporting information can be downloaded at: https://www.mdpi.com/article/10.3390/nu17050909/s1, File S1: Questionnaire used in the study; File S2: Table S1: Univariate analysis for the willingness to eat regularly edible insects as an outcome; Table S2: Univariate analysis for the willingness to eat regularly synthetic meat as an outcome; Table S3: Univariate analysis for the willingness to eat regularly soil-free cultivation products.
