# Supplementary material for: Changing Food in a Changing World: Assessing Compliance to Insects, Cultivated Meat, and Soil-Less Products Among Italian Undergraduates
Source: Nutrients. 2025 Mar 5;17(5):909. doi: 10.3390/nu17050909 (PMC11901983; doi:10.3390/nu17050909)
Supplement: Supplementary file 1 [file nutrients-17-00909-s001.zip › Supplementary File S1. Questionnaire used in the study.pdf]

Supplementary file 1. Questionnaire used in the study

Changing Diet in a Changing World: Assessing Compliance to Insects, Cultivated Meat and Soil-Less Produces among Italian Undergraduates.

Recent years are witnessing the emergence of new food technologies and the spread of novel foods with the aim of increasing the durability of food, replacing food of animal origin or ensuring the production of plants with limited resource consumption. The purpose of the following questionnaire is to investigate Italian citizens' knowledge and readiness for consumption of *novel foods* and foods produced with new technologies.

The questionnaire is completely completed anonymous. In compliance with privacy law, the data collected will be processed in aggregated form, exclusively for research purposes. By selecting this box, you confirm that you are more than 18 years old and have been informed about the characteristics and purpose of the study.

Thank you for your kind cooperation.

Gender

Male

Female

I prefer not to specify it

Age

Only values between 18 and 100 are allowed

What is your nationality?

Italian

Other

Please indicate your mother's highest level of education

Up to lower secondary school (elementary and/or medium)

Upper secondary school diploma (e.g. secondary school, technical/vocational institute)

Degree or more

Please indicate your father's highest level of education

Up to lower secondary school (elementary and/or medium)

Upper secondary school diploma (e.g. secondary school, technical/vocational institute)

Degree or more

Please indicate the university to which you are enrolled

University of Bari

University of Bologna Alma Mater

University of Campania Luigi Vanvitelli

University of Catanzaro Magna Græcia

University of Messina

University of Modena and Reggio Emilia

University of Naples Parthenope

University of Parma

University of Perugia
University of Eastern Piedmont Amedeo Avogadro
University of Rome Foro Italico
University of Rome La Sapienza
University of Turin
Other
Please indicate the level of education you are currently enrolled in
Bachelor’s degree
Master’s degree
Master’s degree
Specialised school
Doctorate
Other post-graduate course
Please indicate your course of study (e.g. Medicine and Surgery, Engineering, etc.)
Do you suffer from a chronic condition? (i.e. hypercholesterolaemia, hypertension, heart disease, diabetes, etc.)
No
Yes
How would you define your current diet? Refer to the eating habits you have generally adopted in the past year
No specific dietary regimen
Vegetarian or vegan
Mediterranean diet (high consumption of plants, fruits, whole cereals and reduced meat consumption)
Diet adapted to particular health conditions (intolerances, diseases, risk conditions)
Energy-restricted diet (low fat or carbohydrate content) for weight loss
Other
Do you use food supplements, even sometimes?
No
Yes

PROPENSITY TO CONSUME NOVEL FOOD: INSECTS, SYNTHETIC MEAT AND PRODUCTS GROWN FROM SOIL-FREE CROPS.

For the purposes of this study, insects are all those edible species authorized to be marketed in some countries, either in whole form or in the form of flour (e.g. grills).

Synthetic meat, also known as cultivated meat, refers to food derived from animal cells (extracted from tissues of chickens, cows or pigs) which are multiplied in vitro, under controlled conditions.

Soil-less crops refer to the production of food obtained by growing plants in controlled environments, without using agricultural land but using other nutrient substrates (e.g. water and mineral salts, peat, coconut fibers, clay, etc.).

Before taking part in this study, have you ever heard about edible insects?

No

Yes

How sustainable do you think insects can be compared to conventional food from animal sources?

Much less

Less

I am not sure

More

Much more

Would you be willing to try edible insects?

definitely no

probably no

unsure

probably yes

definitely yes

Would you regularly consume edible insects?

No

Yes

How much would you be willing to pay to buy edible insects?

Much less than other food of animal origin

Less than other food of animal origin

The same price as other food of animal origin

More than other food of animal origin

Much more than other food of animal origin

Before taking part in this study, have you ever heard about synthetic meat?

No

Yes

How sustainable do you think synthetic meat can be compared to conventional meat?

Much less

Less

I am not sure

More

Much more

Would you be willing to try synthetic meat?

definitely no

probably no

unsure
probably yes
definitely yes
Would you regularly consume synthetic meat?
No
Yes
How much would you be willing to pay to buy synthetic meat?
Much less than conventional meat
Less than conventional meat
The same price as conventional meat
More than conventional meat
Much more than conventional meat
Before taking part in this study, have you ever heard about soil-less crops?
No
Yes
How sustainable do you think products from soil-less crops maybe compared to conventional crops?
Much less
Less
I am not sure
More
Much more
Would you be willing to try products from soil-less crops?
definitely no
probably no
unsure
probably yes
definitely yes
Would you regularly consume products from soil-less crops?
No
Yes
How much would you be willing to pay to buy products from soil-less crops?
Much less than conventional crop products
Less than conventional crop products
The same price as conventional crop products
More than conventional crop products
Much more than conventional crop products
