# Supplementary material for: Changing Food in a Changing World: Assessing Compliance to Insects, Cultivated Meat, and Soil-Less Products Among Italian Undergraduates
Source: Nutrients. 2025 Mar 5;17(5):909. doi: 10.3390/nu17050909 (PMC11901983; doi:10.3390/nu17050909)
Supplement: Supplementary file 1 [file nutrients-17-00909-s001.zip › Supplementary File S2. Results from the univariate analyses.pdf]

Table S1. Univariate analysis for the willingness to eat regularly edible insects as outcome

Variable	Willingness to eat regularly edible insects		p value
	Yes (n=141) n (%)	No (n=1647) n (%)	
**Age mean value ± SD**	25.20 ± 6.32	24.76 ± 7.00	0.023*
**Gender**			
Females	68 (48.23)	1182 (71.77)	<0.001
Males	73 (51.77)	445 (27.02)	
**Body Mass Index**			
Underweight	4 (2.84)	118 (7.16)	
Normal weight	100 (70.92)	1148 (69.7)	
Overweight	28 (19.86)	285 (17.3)	0.246
Obese	9 (6.38)	96 (5.83)	
**Nationality**			
Italian	131 (92.91)	1157 (96.96)	0.010
Other	10 (7.09)	50 (3.04)	
**Mother's educational level**			
Until to secondary school	90 (63.83)	1241 (75.35)	
Degree or postgraduate education	51 (36.17)	406 (24.65)	0.003
**Father's educational level**			
Until to secondary school	102 (72.34)	1347 (81.79)	
Degree or postgraduate education	39 (27.66)	300 (18.21)	0.006
**Geographical area**			
North	33 (23.40)	627 (38.07)	
Center	34 (24.11)	222 (13.48)	<0.001
South	70 (49.65)	697 (42.32)	
**Study Area**			
Medical - health care area	38 (26.95)	532 (32.30)	0.151
Other	103 (73.05)	1,115 (67.70)	
**Chronic disease**			
Yes	16 (11.76)	175 (10.99)	0.781
No	120 (88.24)	1,418 (89.01)	
**Diet regimen**			
Mediterranean Diet	62 (43.97)	558 (33.88)	0.016
Other type of diet	79 (56.03)	1089 (66.12)	
**Use of nutritional supplements**			
Yes	57 (40.43)	657 (39.89)	0.901
No	84 (59.57)	990 (60.11)	

*Mann- Whitney U Test

Table S2. Univariate analysis for the willingness to eat regularly synthetic meat as outcome

Variable	Willingness to eat regularly synthetic meat		p value
	**Yes (n=344)**<br>**n (%)**	**No (n=1444)**<br>**n (%)**	
**Age**<br>**mean value ± SD**	24.77 ± 6.37	24.79 ± 7.09	0.0528*
**Gender**			
Females	211 (61.34)	1,039 (71.96)	0.001
Males	128 (37.21)	390 (27.00)	
**Nationality**			
Italian	332 (96.51)	1,396 (96.67)	0.879
Other	12 (3.49)	48 (3.33)	
**Mother's educational level**			
Until to secondary school	239 (69.48)	1092 (75.62)	0.019
Degree or postgraduate education	105 (30.52)	352 (24.38)	
**Father's educational level**			
Until to secondary school	257 (74.71)	1192 (82.55)	0.001
Degree or postgraduate education	87 (25.29)	252 (17.45)	
**Geographical area**			
North	175 (50.87)	592 (40.99)	0.004
Center	50 (14.53)	206 (14.27)	
South	101 (29.36)	559 (38.69)	
**Study Area**			
Medical - health care area	224 (65.12)	994 (68.85)	0.183
Other	120 (34.88)	450 (31.15)	
**Chronic disease**			
Yes	37 (10.76)	154 (10.99)	0.881
No	291 (84.59)	1,247 (89.01)	
**Diet regimen**			
Mediterranean Diet	127 (36.92)	493 (34.15)	0.331
Other type of diet	127 (36.92)	951 (65.86)	
**Use of nutritional supplements**			
Yes	149 (43.31)	565 (39.14)	0.154
No	195 (56.69)	879 (60.86)	

*Mann- Whitney U Test

Table S3. Univariate analysis for the willingness to eat regularly soil-free cultivation products

Variable	Willingness to eat regularly soil-free cultivation products		p value
	**Yes (n=472)**<br>**n (%)**	**No (n=1316)**<br>**n (%)**	
**Age**<br>**mean value ± SD**	25.726 ± 7.4	24.45 ± 6.757	<0.001*
**Gender**			
Females	316 (67.00)	934 (70.97)	0.170
Males	152 (32.20)	366 (27.81)	
**Nationality**			
Italian	451 (95.56)	1,277 (97.04)	0.124
Other	21 (4.44)	39 (2.96)	
**Mother's educational level**			
Until to secondary school	325 (68.86)	1006 (76.44)	0.001
Degree or postgraduate education	147 (31.15)	310 (23.55)	
**Father's educational level**			
Until to secondary school	366 (77.54)	1083 (82.29)	0.011
Degree or postgraduate education	106 (22.46)	233 (17.72)	
**Geographical area**			
North	243 (51.48)	524 (39.81)	<0.001
Center	62 (13.14)	194 (14.74)	
South	141 (29.87)	519 (39.42)	
**Study Area**			
Medical - health care area	324 (68.64)	894 (67.94)	0.776
Other	148 (31.36)	422 (32.06)	
**Chronic disease**			
Yes	53 (11.45)	138 (10.85)	0.676
No	405 (88.55)	1,133 (89.15)	
**Diet regimen**			
Mediterranean Diet	189 (40.04)	431 (32.75)	0.004
Other type of diet	283 (59.96)	885 (67.25)	
**Use of nutritional supplements**			
Yes	192 (40.68)	522 (39.66)	0.700
No	280 (59.32)	794 (60.34)	

*Mann- Whitney U Test
